# Regulation of NK Cell Activation and Effector Functions by the IL-12 Family of Cytokines: The Case of IL-27

**DOI:** 10.3389/fimmu.2017.00025

**Published:** 2017-01-19

**Authors:** Norberto Walter Zwirner, Andrea Ziblat

**Affiliations:** ^1^Laboratorio de Fisiopatología de la Inmunidad Innata, Instituto de Biología y Medicina Experimental (IBYME, CONICET), Ciudad de Buenos Aires, Argentina; ^2^Facultad de Ciencias Exactas y Naturales, Departamento de Química Biológica, Universidad de Buenos Aires, Ciudad de Buenos Aires, Argentina

**Keywords:** NK cells, cytokines, dendritic cells, cytotoxicity, immunologic, innate immunity

## Abstract

Natural killer (NK) cells are characterized by their ability to detect and induce apoptosis of susceptible target cells and by secretion of immunoregulatory cytokines such as IFN-γ. Activation of these effector functions is triggered upon recognition of tumor and pathogen (mostly virus)-infected cells and because of a bidirectional cross talk that NK cells establish with other cells of myeloid origin such as dendritic cells (DC) and macrophages. A common characteristic of these myeloid cells is their ability to secrete different members of the IL-12 family of cytokines such as IL-12, IL-23, and IL-27 and cytokines such as IL-15 and IL-18. Although the effect of IL-12, IL-15, and IL-18 has been characterized, the effect of IL-23 and IL-27 on NK cells (especially human) remains ill-defined. Particularly, IL-27 is a cytokine with dual functions as it has been described as pro- and as anti-inflammatory in different experimental settings. Recent evidence indicates that this cytokine indeed promotes human NK cell activation, IFN-γ secretion, NKp46-dependent NK cell-mediated cytotoxicity, and antibody (Ab)-dependent NK cell-mediated cytotoxicity (ADCC) against monoclonal Ab-coated tumor cells. Remarkably, IL-27 also primes NK cells for IL-18 responsiveness, enhancing these functional responses. Consequently, IL-27 acts as a pro-inflammatory cytokine that, in concert with other DC-derived cytokines, hierarchically contributes to NK cells activation and effector functions, which likely contributes to foster the adaptive immune response in different physiopathological conditions.

## Introduction

Natural killer (NK) cells constitute one of the three major lymphoid cell populations in blood. They play a protective role against viral infections and tumors, although additional evidence indicates that NK cells are also key players during immunity against other intracellular pathogens (1, 2). In humans, evidence about their role during viral infections came from the observation that patients with rare primary immunodeficiencies that lead to the absence of NK cells or the presence of dysfunctional NK cells display increased susceptibility to different viruses (3, 4). Currently, we know that the relevance of NK cells in immunity goes far beyond viral infections, being active immunoregulatory cells during infections with other pathogens, and also during autoimmune processes and in allograft rejection (1). Moreover, it has been established that NK cells are abundant in different tissues where they may exert such functions, in particular, immunosurveillance against pathogens (5, 6).

From a functional aspect, human and mouse NK cells share the ability to induce apoptosis of susceptible target cells through the secretory and death receptor-mediated pathways (FasL and TRAIL) as well as the capacity to secrete immunoregulatory cytokines (7). Nevertheless, phenotypic characterization of NK cells in both species is quite different. In mice, NK cells are mostly identified as CD3^−^ CD49b^+^ cells, although in C57BL/6 mice but they can also be characterized as CD3^−^CD161b/CD161c^+^ cells (better known as CD3^−^NK1.1^+^ cells) (2, 8). In humans, NK cells are characterized as CD3^−^CD56^+^ cells but can be subdivided into different subpopulations based on a differential expression of CD56 and CD16 (9, 10). The majority of peripheral blood NK cells (about a 90%) are CD3^−^CD56^dim^CD16^+^, which display a high content of perforin and granzymes and a strong cytotoxic activity, while the rest of NK cells in blood are CD3^−^CD56^bright^CD16^dim/−^ and produce immunoregulatory cytokines in response to different stimuli (11, 12). This subpopulation of NK cells is highly abundant in second lymph organs, where they instruct dendritic cells (DC) to promote Th1- and cytotoxic CD8 T cell-biased responses, shaping in this way the adaptive immunity (13, 14). Some evidence suggests that CD56^bright^CD16^dim/−^ can differentiate into CD56^dim^CD16^+^ cells upon *in vitro* stimulation, indicating that they may constitute developmental stages of fully mature CD56^dim^CD16^+^ NK cells (15–17). NK cell subpopulations also express different chemokine receptors involved in their homing to different anatomical niches (5, 18).

Recently, identification of innate immune lymphoid cell populations (ILC), especially in mucosal sites, led to a reclassification of NK cells as members of this extended family of cells of the innate immune response (19–22). ILC contribute to tissue homeostasis, and they seem to be important players of immunity in mucosal sites. Three groups of ILC populations have been described (ILC1, ILC2, and ILC3), which differ in their transcriptional, phenotypic, and transcriptional signatures, respectively (19, 21, 22). Moreover, ILC phenotype and function mirrors the phenotype and function of T cells, indicating that innate immune cells display a similar functional compartmentalization as occurs with adaptive immune cells. NK cells have been classified as a subgroup of ILC1, suggesting that they could be some sort of ancestors or innate counterparts of T helper 1 and cytotoxic T lymphocyte (CTL) cells (19, 21, 22). Although all ILC1 express T-bet, respond to IL-12 and IL-15 and share the ability to produce IFN-γ, only NK cells express EOMES, which differentiates them from other ILC1 populations (19, 21, 22).

A vast array of surface receptors confer NK cells the ability to sense their environment. Direct recognition of target cells through inhibitory and activating receptors is a critical event that determines activation of NK cell-mediated cytotoxicity against susceptible cells (virus-infected or neoplastic cells), preserving healthy cells from such response (7). Many receptors that recognize discrete ligands expressed on target cells and that trigger NK cell activation or promote inhibition of NK cell-mediated effector functions have been identified and cloned (2, 10). The better characterized receptors that regulate target cell recognition and activation by NK cells are CD16 or FcRγIII [which mediates antibody (Ab)-recognition of target cells and triggers Ab-dependent cell-mediated cytotoxicity or ADCC], CD314 or NKG2D, the natural cytotoxicity receptors CD335 (NKp46), CD336 (NKp44) and CD337 (NKp30), CD226 (DNAM-1), CD244 (2B4), members of the CD158 or killer immunoglobulin-like receptor (KIR) family that carry a short cytoplasmic tail (KIR2DS and KIR3DS) and CD94/NKG2C, among others (2, 10, 23). Conversely, inhibitory receptors that preclude NK cell activation are members of the CD158 or KIR family that carry a long cytoplasmic tail (KIR2DL and KIR3DL), CD94/NKG2A, TIGIT, and CD85j (ILT-2, LILRB1, or LIR-1), among others (2, 10, 23).

Natural killer cells not only sense and respond to ligands expressed on the cell surface of target cells. Instead, functional response of NK cells also depends on recognition of soluble factors such as pro-inflammatory cytokines (24). Nonetheless, other soluble factors also exert immunoregulatory functions on these cells. We and others (25–30) observed that NK cells express endosomal toll-like receptors (TLRs) and respond to specific agonists. In particular, human NK cells express functional TLR3, TLR7, and TLR9, and stimulation of NK cells with their agonists triggers IFN-γ secretion only in the presence of suboptimal concentrations of IL-12 or IFN-α but not IL-15 (25). This effect was further potentiated by co-engagement of NKG2D, one of the major cell surface receptors involved in recognition and elimination of tumor cells by NK cells, but TLR agonists do not seem to exert immunoregulatory effects on NKG2D-dependent NK cell-mediated cytotoxicity (5). Therefore, NK cells can sense and integrate signals derived from their surrounding environment, and that are detected by different categories of receptors.

Biological functions of NK cells are tightly regulated during their interaction with DC as a consequence of which NK cells promote maturation of DC and become activated by cell surface receptors such as NKp30 (31) and DNAM-1 (32) and cytokines such as IL-12, IL-15, and IL-18 (9, 13, 31–35). Remarkably, the consequences of this interaction are not only manifested in NK cells but also impact on the adaptive immunity as NK cells promote maturation of DC and instruct them to shape T cell activation toward Th1- and CTL-mediated responses (13, 14, 31, 33).

In this context, an integral analysis of factors that regulate NK cell effector functions may contribute to the development of novel strategies to improve immunosurveillance and promote a sustained tumoricidal capacity of NK cells (7). Therefore, the focus of our laboratory has been the investigation of how NK cells sense their environment and unravel novel factors that affect their phenotype and functions.

## Regulation of NK Cell Activation and Effector Functions by IL-27

IL-12 is the first described member of an extended family of cytokines produced mostly by myeloid cells (DC and macrophages) in response to infectious agents and other insults (36). IL-12 promotes the generation of Th1, IFN-γ-producing cells during naive CD4^+^ T cell activation (37). Also, IL-12 produced by macrophages triggers NK cell-mediated IFN-γ production during infection with intracellular parasites (38) and contributes to protection during acute infection (39). These findings unraveled the existence of a cytokine axis in which myeloid cell-derived IL-12 triggers lymphoid cell-derived IFN-γ production and contributes to resistance to infection.

Members of the IL-12 family of heterodimeric cytokines share protein subunits and receptor chains. IL-12 is composed by two subunits, namely, p35 and p40, and is recognized by a heterodimeric receptor composed of two chains IL-12Rβ1 and IL-12Rβ2 (36). Signaling through this receptor activates mainly STAT4, activates T-bet, and leads to IFN-γ production in NK and T cells, thus mediating pro-inflammatory effects (40–42). Since naïve T cells do not express IL-12Rβ2, IL-12 alone does not seem to be sufficient to guide T cell activation toward Th1 cells (43). A similar effect was described for IFN-γ production by human and mouse NK cells (25, 44–46), suggesting that IL-12 requires a cooperation with other factors to properly exert its effects on NK and T cell-derived IFN-γ production. IL-12 also enhances NK cell-mediated cytotoxicity against different target cells, affects expression of some cell surface receptors involved in target cell recognition (47–49), and, more recently, IL-12 has also been involved in the generation of memory-like NK cells (50, 51). IL-12 is secreted by DC and macrophages and has been shown to be a major player of a bidirectional cross talk that they establish with NK or T cells (36). Also, as a consequence of their cross talk with macrophages, NK cells can stimulate production of nitric oxide (NO) due to upregulation of inducible NO synthase (52). In addition, as a consequence of their cross talk with DC, NK cells can promote upregulation of costimulatory molecules such as CD86 (53).

Other members of the IL-12 family of cytokines are IL-23, IL-27, and IL-35 (36, 54) which, as mentioned, are heterodimeric proteins that share not only one subunit with another member of the family but also signal through heterodimeric receptors with shared subunits (54, 55). As with IL-12, macrophages and DC can produce IL-23 and IL-27 upon sensing pathogens or their products (56–59). IL-23 is composed by one subunit shared with IL-12 (p40) that is associated with the p19 subunit to constitute the active form of IL-23. This cytokine signals through a heterodimeric receptor composed of IL-12Rβ1 and IL-23R, which activates Jak2/Tyk2, STAT1/STAT3/STAT4/STAT5 (60). IL-23 activates NK cells and in this way, contributes to the antitumor immune response (61, 62). Nevertheless, other authors failed to demonstrate an effect of IL-23 on NK cells (63, 64), making the effects of this cytokine on NK cells an open question that warrants further investigation.

**Figure 1:**
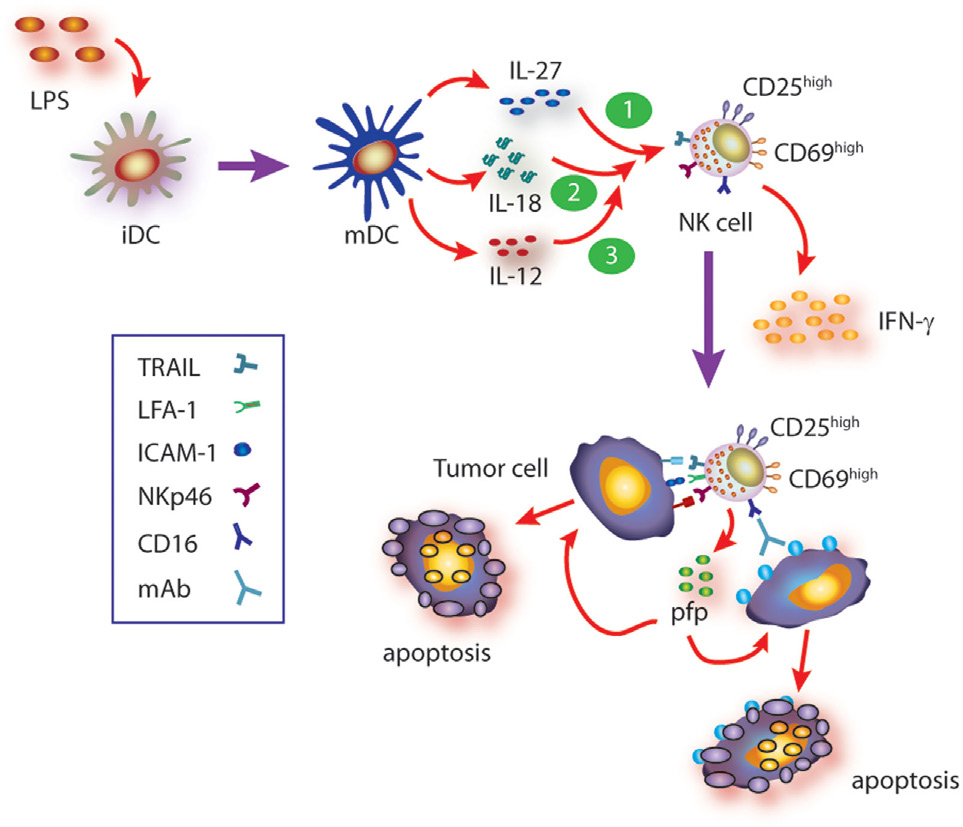
**Summary of cytokine axis involving IL-27 in human natural killer (NK) cell activation**. Mature DC (mDC) secrete IL-12, IL-27, and IL-18, among others. During the cross talk between mDC and NK cells (and besides the known effect of IL-12), IL-27, alone or in concert with IL-18, triggers NK cell activation (upregulation of CD25 and CD69), IFN-γ production, and cytotoxicity against target cells that are otherwise resistant to non-stimulated NK cells. Such cytotoxic response involves recognition of target cells through NKp46- and CD16-dependent mechanisms (ADCC) and induction of target cell apoptosis *via* granule exocytosis and TRAIL-mediated mechanisms. Moreover, IL-27 also primes NK cells for IL-18-mediated augmented IFN-γ secretion which in turn upregulates ICAM-1 on target cells, facilitates the formation of NK cell–target cell conjugates, and therefore further increases the cytotoxic activity of NK cells. Since IL-18 primes NK cells for IL-12 responsiveness, IL-27-driven priming of NK cells for IL-18 may also contribute to further potentiate IL-12 responsiveness and fostering NK cell effector functions.

IL-27, in turn, is a heterodimeric cytokine composed by the EBI3 and p28 subunits that signals through a heterodimeric receptor composed by the WSX-1 and CD130/gp130 chains (54, 55, 65, 66). As with other members of this familty of cytokines, IL-27 is produced mainly by DC and macrophages upon microbial insults (55). Paradoxically, IL-27 displays pro- and anti-inflammatory functions due to activation of STAT1 and STAT3, respectively (36), but its pro-inflammatory effects depend on induction of T-bet and IL-12Rβ2 expression (67–69). In line with a dual role of IL-27, it has been shown that this cytokine prevents tissue damage induced by excessive inflammation (54, 70). The effect of IL-27 on NK cells and their ability to control tumor growth have been described in some mouse models (63, 70–76), while in other tumor models, an effect of IL-27 on NK cells was not observed (77). Therefore, the effects of IL-27 on mouse NK cells might be tumor-type dependent. In humans, it was reported that IL-27 can costimulate NK cells for IFN-γ gene expression (78), while we observed that mature DC secrete IL-27 and that this cytokine contributes to NK cell activation and effector functions (79). Indeed, IL-27 can directly trigger IFN-γ secretion through activation of STAT1 and promote activation of NK cells (upregulation of CD25 and CD69). IL-27 also promotes upregulation of NKp46 and subsequent NKp46-dependent NK cell-mediated cytotoxicity against target cells that are otherwise resistant to NK cell-mediated cytotoxicity, through the secretory pathway and TRAIL (79). IL-27 also potentiates ADCC induced by therapeutic monoclonal antibodies such as rituximab, trastuzumab, and cetuximab, suggesting that IL-27 may be helpful as adjuvant during immunotherapy in human patients (79). The effects of IL-27 on NK cells are summarized in Figure 1.

## Cooperation Between Cytokines for NK Cell Stimulation: The Case of IL-27 and IL-18

Cooperative effect of cytokines, in particular those secreted by DC and macrophages, has been described for many of them and reviewed elsewhere (24). Briefly, cooperative effects of IL-12 and IL-2 or IL-15 (80, 81), IL-2 and IL-15 (82), IL-12 and IL-18 (81, 83–85) for NK cell activation, IFN-γ production, and cytotoxicity have been described. In most cases, underlying mechanisms of cytokine cooperation for NK cell activation remain ill-defined. IL-18 belongs to the IL-1 superfamily and has the peculiarity of having a critical effect on NK cells (86). IL-18 seems to play a major role as “cooperating cytokine” for NK cell activation and elicitation of effector functions (87, 88). Accordingly, NK cells from IL-18^−/−^ mice display a deep impaired immune response against tumors and cannot be properly stimulated *in vivo* with IL-12 to secrete IFN-γ (89). These and other experimental results led to the notion that IL-18 actually primes NK cells to become responsive to IL-12 (89–91). Remarkably, we demonstrated that IL-27 also primes NK cells but for IL-18-mediated IFN-γ secretion inducing upregulation of T-bet expression in NK cells (79). T-bet is a critical transcription factor that regulates IFN-γ production (92, 93) by promoting IFN-γ gene transcription (94). Moreover, cooperation between IL-27 and IL-18 enhances NK cell-mediated cytotoxicity through the secretory pathway and TRAIL and involves NK cell-derived IFN-γ. This is because IFN-γ secretion during effector–target cell contact increases the percentage of ICAM-1^+^ target cells that in turn facilitates the formation of NK cell–target cell conjugates and delivery of the cytotoxic hit (95). These effects are summarized in Figure 1.

As DC and macrophages stimulated with microbial products or tumor cells can secrete IL-12, IL-18, and IL-27 (36, 73), it is possible that stimulatory effects of IL-27 may occur when DC or macrophages secrete this cytokine and establish a bidirectional cross talk with NK cells. During this cross talk, IL-27 may prime NK cells for IL-18 responsiveness, while IL-18 secreted at the synaptic cleft between NK cells and DC (91) may in turn prime NK cells for IL-12 responsiveness (90). Although a kinetic analysis of the production of these cytokines needs to be performed to establish the temporal relationship in their secretion, the cooperation between IL-27 and IL-18 that we described unravels the existence of a hierarchical cytokine network that is relevant during DC-NK cell cross talk that generates fully functional NK cells. In line with this hierarchical cytokine network in NK cell activation is the fact that IL-27 can initiate Th1 development by naïve T cells by promoting activation of STAT1 and STAT3, expression of T-bet, repression of GATA3 (involved in Th2 differentiation), and production of IL-12Rβ2 chain (67, 68, 96, 97). These changes in CD4 T cells during activation confer them the ability to sense DC-derived IL-12 and consequently follow the path of Th1 differentiation, leading to secretion of IFN-γ.

Collectively, the cytokine axis composed of IL-27/IL-18/IL-12 is indeed involved in optimal NK cell activation and in skewing CD4 T cell responses through a cross talk between these lymphoid cells (NK cells and T cells) and myeloid cells (DC), representing an important link between innate and adaptive immunity.

## Concluding Remarks

Natural killer cells are currently viewed not only as cytotoxic cells but also as strong producers of immunoregulatory cytokines, in particular, IFN-γ. They belong to the family of ILC, and their effector functions are tightly regulated by interaction with DC and other cells of myeloid lineage, which secrete cytokines with NK cell-stimulating activity. IL-12 is one of the most relevant cytokines produced by myeloid cells that promote NK cell activation. The discovery of other members of the IL-12 family of cytokines, such as IL-23 and IL-27, and exploration of cooperation between cytokines for NK cell activation have established that NK cells also become activated by IL-27. Interestingly, IL-27 not only exerts direct effects on NK cells but also primes them for IL-18-responsiveness, which unveils another aspect of the intricate cytokine network that regulates NK cell biological functions and that further demonstrates a hierarchical effect of different cytokines on these cells. Consequently, NK cells display the ability to integrate multiple signals from their environment and adjust their effector functions accordingly, probably to optimize the magnitude of their response to pathogens and tumor cells and shape adaptive immunity in different physiopathological conditions.

## Author Contributions

NZ designed and wrote the review and prepared the figures. AZ revised the manuscript and figures.

## Conflict of Interest Statement

The authors declare that the research was conducted in the absence of any commercial or financial relationships that could be construed as a potential conflict of interest.
